# Three-dimensional virtual model for robot-assisted partial nephrectomy: a propensity-score matching analysis with a contemporary control group

**DOI:** 10.1007/s00345-024-05043-9

**Published:** 2024-05-20

**Authors:** Antonio Andrea Grosso, Fabrizio Di Maida, Luca Lambertini, Anna Cadenar, Simone Coco, Elena Ciaralli, Vincenzo Salamone, Gianni Vittori, Agostino Tuccio, Andrea Mari, Giuseppe Mario Ludovico, Andrea Minervini

**Affiliations:** 1 Department of Experimental and Clinical Medicine, Minimally-Invasive Urology and Andrology, University of Florence—Unit of Oncologic, Careggi Hospital, San Luca Nuovo, 50134 Florence, Italy; 2 Department of Urology, “F. Miulli” General Hospital, Acquaviva Delle Fonti, Bari, Italy

**Keywords:** Augmented reality, Partial nephrectomy, Renal cancer, Robotic surgery, Three-dimensional models

## Abstract

**Purpose:**

To compare two cohorts of patients submitted to robot-assisted partial nephrectomy (RAPN) with vs without the use of three-dimensional virtual models (3DVMs).

**Methods:**

We screened a prospective consecutive cohort of 152 patients submitted to RAPN with 3DVM and 1264 patients submitted to RAPN without 3DVM between 2019 and 2022. Propensity score matching analysis (PSMA) was applied. Primary endpoint was to evaluate whereas RAPNs with 3DVM were superior in terms of functional outcomes at 12-month. Secondary endopoints were to compare perioperative and oncological outcomes. Multivariable logistic regression analyses (MVA) tested the associations of clinically significant eGFR drop and 3DVMs. Subgroups analysis was performed for PAUDA-risk categories.

**Results:**

100 patients for each group were analyzed after PSMA. RAPN with 3DVM presented a higher rate of selective/no clamping procedure (32% vs 16%, *p* = 0.03) and a higher enucleation rate (40% vs 29%, *p* = 0.04). As concern to primary endopoint, 12-month functional preservation performed better within 3DVM group in terms of creatinine serum level (median 1.2 [IQR 1.1–1.4] vs 1.6 [IQR 1.1–1.8], *p* = 0.03) and eGFR (median 64.6 [IQR 56.2–74.1] vs 52.3 [IQR 49.2–74.1], *p* = 0.03). However, this result was confirmed only in the PADUA ≥ 10 renal masses. Regarding secondary endpoints, no significative difference emerged between the two cohorts. MVA confirmed 3DVM as a protective factor for clinically significant eGFR drop only in high-risk (PADUA ≥ 10) masses.

**Conclusions:**

RAPN performed with the use of 3DVM assistance resulted in lower incidence of global ischemia and higher rate of enucleations. The positive impact of such technology was found at 12-month only in high-risk renal masses.

**Supplementary Information:**

The online version contains supplementary material available at 10.1007/s00345-024-05043-9.

## Introduction

In contemporary clinical practice, partial nephrectomy (PN) has emerged as the preferred standard for the surgical management of clinical T1 renal neoplasms [1]. This approach offers several advantages over radical nephrectomy, notably in terms of preserving renal function while maintaining a comparable level of safety regarding oncological outcomes [2–4]. In recent times, the increasing adoption of robotic-assisted techniques has expanded the application of PN to even complex or larger renal masses [5, 6]. This has allowed for the optimization of surgical outcomes that influence renal function impairment, including factors such as vascular clamping-related ischemia, the removal of perilesional healthy tissue, and suture-related ischemia.

In this pursuit of improved surgical outcomes, innovative imaging technologies have been explored, such as highly accurate three-dimensional virtual models (3DVMs) [7, 8]. It is worth noting that, despite 3DVMs have been tested in terms of perioperative and functional outcomes following robot-assisted partial nephrectomy (RAPN) [9, 10], the available literature is still lacking and demand for further evidence.

The aim of the present study was to conduct a comparative analysis between two contemporary cohorts of patients submitted to RAPN with vs without the use of 3DVMs trying to demonstrate if 3DVMs could impact on oncologic and functional outcomes following RAPN, after applying a propensity-score matching analysis (PSMA) and incorporating different tumor excision techniqes.

## Materials and methods

### Population data

In the present study, all patients with radiological suspected renal mass who were candidates for robotic PN were prospectively considered between 2019 and 2022. For inclusion in the study patients were required to undergo four-phase contrast-enhanced CT and 3D PDF models were obtained in collaboration with a team of biomedical engineers (MEDICS, Turin). Data of this population were compared with a prospective control cohort of patients treated by robotic PN during the same time-period, but without 3DVMs.

Metastatic patients, as well as patients presenting pathological lymph nodes enlarged and/or bilateral and/or multiple tumors were excluded from the final analysis. Preoperative patient’s characteristics were recorded. All tumors were scored according to the Preoperative Aspects and Dimensions Used for an Anatomical (PADUA) nephrometric classification of renal masses [11]. Peri- and postoperative data were thoroughly gathered, including ischemia strategy (on-clamp vs selective clamping vs off-clamp), operative time, warm ischemia time and complication rate. Complications were graded according to Clavien-Dindo (CD) classification [12]. Tumor stage was classified according to the 2010 TNM criteria [13]. Renal function was measured at baseline, discharge and scheduled follow-up visits as creatinine level and estimated glomerular filtration rate (eGFR), using the Chronic Kidney Disease Epidemiology Collaboration (CKD-EPI) equation [14]. For the study purpose Chronic kidney disease (CKD) stage was assessed according to the Kidney Disease Improvement Global Outcomes—KDIGO criteria [15]. Resection technique was visually classified by the surgeon as enucleation (SIB score 0–2), enucleoresection (SIB score 3–4) or resection (SIB score 5) according to the SIB score, as previously reported [16].

### Surgical data

RAPNs were performed by three highly-experienced robotic surgeons with > 500 robotic surgeries executed so far. The institution where the study was conducted represents a tertiary referral center for minimally-invasive treatment of kidney cancer and is equipped with adequate technology as well as dedicated surgical, anesthesiologist and nursing team. Surgical procedure steps are discussed in previous publications [17]. Briefly, The Da Vinci Si system was used in all the cases, (Intuitive Surgical, Sunnyvale, CA, USA), in a three-arm configuration with a 30° laparoscope optic. After medialized the colon, the renal pedicle is identified and isolated. Once the tumor template has been marked with monopolar cautery, the choice and type of clamping is based on tumor and patient features (i.e. tumor complexity and vasculature, baseline kidney function). Then, the tumor is removed adopting a tailored excision technique (resection, enucleoresection, enucleation). Finally, renorraphy is always performed separately with a medullary and cortical suture. Eventually, hemostatic agents are placed on tumor resection bad. Peri- and postoperative data were thoroughly gathered as previously mentioned.

### Endpoints

Our primary endpoint was to evaluate whereas RAPNs with 3DVM were superior in terms of functional outcomes defined as 12-month creatinine serum level and 12-month eGFR in the overall cohort and in subgroup analysis. Moreover, we tested the association of 3DVMs and clinically significant eGFR drop defined as worsening of at least 30% of postoperative renal function (eGFR loss ≥ 30% from baseline value) [18]. Secondary endpoints were to compare perioperative and oncological outcomes. For the first, we considered warm ischemia time, intraoperative and postoperative complications as well as hospitalization time, while for the latest positive surgical margin (PSM) and local recurrence rate were evaluated.

### Statistical analysis

Categorical, continuous parametric and not-parametric variables were reported as frequencies and proportions, mean and standard deviation (SD) or as median and interquartile range (IQR), respectively. Unpaired *T* Test, Mann–Whitney and Pearson’s chi-square tests were used to compare variables, as appropriate. To limit the potential effect of selection bias, we generated a 1:1 propensity score–matched cohort (nearest-neighbour PSMA using a caliper width of 0.1 of the standard deviation of the logit of the propensity score). Matching variables represented Charlson Comorbidity Index (CCI), PADUA score, and baseline creatinine serum level. Furthermore, we executed a subgroup analysis stratifying the results according to PADUA risk categories (PADUA 6–7 Low-risk; PADUA 8–9 Intermediate-risk; PADUA ≥ 10 High-risk). Finally, univariable and multivariable (MVA) logistic regression analyses tested the associations of clinically significant eGFR drop and 3DVMs. To account for potential different impacts of 3DVMs on the probability of clinically significant eGFR drop, MVA was adjusted for further variables including resection technique, CCI and warm ischemia time. Statistical analyses were performed using STATA^®^16 software (StataCorp LLC, US). All tests were two sided, and statistical significance level was determined at *p* < 0.05.

## Results

Overall, 152 RAPNs with 3DVM and 1264 RAPNs without 3DVMs were screened. After applying propensity-score matching analysis, finally 100 cases for both groups were considered eligible for analysis.

Patients and tumor characteristics were comparable between both groups in terms of preoperative and tumor features (Table 1). Table 2 showed perioperative and postoperative outcomes. Pedicle management was the only variable differing among the two cohorts since global ischemia was adopted in the vast majority of patients treated with RAPN without 3DVM (84% vs 68%, *p* = 0.03). Warm ischemia time, intraoperative and postoperative complications, as well as hospitalization time did not differ between the cohorts (*p* > 0.05). No differences were found in terms of creatinine serum level (median 1.1 [IQR 0.9–1.3] vs 1.2 [1.0–1.3], *p* = 0.51) and eGFR (69.3 [61.2–77.1] vs 67.1 [61.0–75.8], *p* = 0.22) at discharge.

**Table 1:** Preoperative patient’s and tumor’s features of the two population groups after propensity-score matching analysis

		3DVM (*n* = 100)	No 3DVM (*n* = 100)	*p* value
Gender, *n* (%)	Male	59 (59.0)	56 (56.0)	0.11
	Female	41 (41.0)	44 (44.0)	
Age (years), median (IQR)		68 (60–74)	67 (62–75)	0.21
BMI (kg/m^2^), median (IQR)		25.4 (22–27.6)	25.1 (21.8–26.9)	0.34
CCI age-adjusted, median (IQR)		3 (2–4)	3 (2–4)	0.28
Clinical tumor size (mm), median (IQR)		44 (29–68)	41 (31–66)	0.67
cT stage, *n* (%)	cT1a	21 (21.0)	26 (26.0)	0.55
	cT1b	54 (54.0)	48 (48.0)	
	cT2a	25 (25.0)	26 (26.0)	
PADUA score, median (IQR)		9 (7–11)	9 (7–11)	0.18
PADUA risk-category, *n* (%)	Low-risk (6–7)	22 (22.0)	23 (23.0)	0.66
	Intermediate-risk (8–9)	41 (41.0)	39 (39.0)	
	High-risk (≥ 10)	37 (37.0)	38 (38.0)	
Hemoglobine blood level (g/dL), median (IQR)		14.1 (13.6–15.8)	14.5 (13.1–15.6)	0.17
Creatinine serum level (mg/dL), median (IQR)		0.9 (0.8–1.1)	1.0 (0.8–1.1)	0.34
eGFR (mL/min), median (IQR)		77.1 (65.3—82.7)	74.5 (63.1—87.4)	0.25
CKD stage according to KDIGO criteria, *n* (%)	G1 (eGFR ≥ 90)	28 (28.0)	22 (22.0)	0.41
	G2 (eGFR 60—89)	49 (49.0)	51 (51.0)	
	G3 (eGFR 30—59)	16 (16.0)	18 (18.0)	
	G4 (eGFR 16–29)	7 (7.0)	9 (9.0)	
	G5 (eGFR ≤ 15)	0 (0.0)	0 (0.0)	

*BMI* Body Mass Index, *CCI* Charlson Comorbidity Index, *CKD* Chronic Kidney Disease, eGFR Estimated Glomerular Filtration Rate

**Table 2:** Perioperative and postoperative surgical outcomes of the two population groups after propensity-score matching analysis

		3DVM (*n* = 100)	No 3DVM (*n* = 100)	*p* value
Surgical access, *n* (%)	Transperitoneal	97 (97.0)	95 (95.0)	0.28
	Retroperitoneal	3 (3.0)	5 (5.0)	
Operative time (min), median (IQR)		90 (73–120)	97 (75–120)	0.46
Pedicle management, *n* (%)	Global Ischemia	68 (68.0)	84 (84.0)	0.03
	Selective clamping	21 (21.0)	9 (9.0)	
	Clampless	11 (11.0)	7 (7.0)	
Global ischemia time (min), median (IQR)		15 (11–19)	15 (11–21)	0.19
Estimated blood loss (mL), median (IQR)		100 (50—200)	100 (50–200)	0.34
Intraoperative complication, *n* (%)		0 (0.0)	1 (1.0)	0.28
Postoperative complication, *n* (%)	Clavien-Dindo I	5 (5.0)	6 (6.0)	0.19
	Clavien-Dindo II	7 (7.0)	6 (6.0)	
	Clavien-Dindo III	2 (2.0)	3 (3.0)	
Hospitalization time (days), median (IQR)		3 (3–4)	3 (3–4)	0.26
Hemoglobine blood level at discharge (g/dL), median (IQR)		12.9 (12.0–13.7)	13.0 (11.9–13.7)	0.37
Creatinine serum level at discharge (mg/dL), median (IQR)		1.1 (0.9–1.3)	1.2 (1.0–1.3)	0.51
eGFR at discharge, (mL/min), median (IQR)		69.3 (61.2–77.1)	67.1 (61.0–75.8)	0.22
Readmission, *n* (%)		1 (1.0)	1 (1.0)	0.18
Pathological T-stage, *n* (%)	pT1a	18 (18.0)	21 (21.0)	0.19
	pT1b	49 (49.0)	41 (41.0)	
	pT2a	20 (20.0)	19 (19.0)	
	pT2b	0 (0.0)	2 (2.0)	
	pT3a	13 (13.0)	17 (17.0)	
Pathological tumor diameter (mm), median (IQR)		46 (27–70)	44 (29–71)	0.31
Positive surgical margin, *n* (%)		3 (3.0)	6 (6.0)	0.05
SIB score, *n* (%)	Resection (≥ 5)	17 (16.0)	18 (18.0)	0.04
	Enucleoresection (3–4)	44 (44.0)	53 (53.0)	
	Enucleation (0–2)	40 (40.0)	29 (29.0)	
Local Recurrence rate, *n* (%)		2 (2.0)	1 (1.0)	0.27
12-month creatinine serum level (mg/dL), median (IQR)		1.2 (1.1–1.4)	1.6 (1.1–1.8)	0.03
12-month eGFR (mL/min), median (IQR)		64.6 (56.2–74.1)	52.3 (49.2–74.1)	0.03
Clinical significant eGFR drop, *n* (%)		17 (17.0)	27 (27.0)	0.03
Follow-up, median (IQR)		12 (12–19)	27 (19—36)	0.01

Clinical significant eGFR drop: worsening of at least 30% of postoperative renal function (eGFR loss > 30% from baseline value)

*eGFR* Estimated Glomerular Filtration Rate

In terms of postoperative features, a higher percentage of enucleation in the 3DVM group was found using the SIB score, as compared to the control group (40% vs 29%, *p* = 0.04). On the other hand, local recurrence (2% vs 1%, *p* = 0.27) and positive surgical margins (3% vs 6%, *p* = 0.05) rate were comparable between the cohorts. Median 12-month creatinine serum level (1.2 [IQR 1.1–1.4] vs 1.6 [IQR 1.1–1.8] *p* = 0.03) was significantly lower in the 3DVM group while median eGFR was statistically higher as compared to the control group (64.6 [IQR 56.2–74.1] vs 52.3 [IQR 49.2–74.1], *p* = 0.03).

**Fig. 1:**
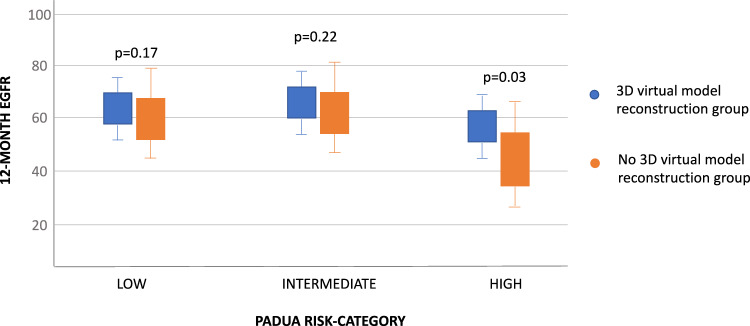
Box-plot depicting comparative values of eGFR between RAPN with/without 3DVM stratified for PADUA-risk categories

When stratifying for PADUA risk category (Fig. 1), a 12-month functional benefit was confirmed only in the high-risk subgroup (median eGFR 58.2 [IQR 50.1–62.4] vs 43 [IQR 37.8–52.1], *p* = 0.03).

Finally, at MVA, 3DVM was found to be protective for clinically significant eGFR drop (Odd Ratio 1.65 [95% Confidence Interval 1.31–2.89]) only in the subgroup of PADUA ≥ 10 masses (Table 3).

**Table 3:** Multivariate logistic regression analysis performed after propensity-score matching analysis for clinically significant eGFR drop and stratified for PADUA risk-categories

	Odd ratio	95% Confidence interval	*P* value
High-complexity renal masses (Padua ≥ 10)			
3D virtual model reconstruction *(vs no reconstruction)*	0.65	(0.47–0.89)	0.02
Enucleation *(vs enucleoresection)*	0.74	(0.39–1.91)	0.11
Enucleation *(vs resection)*	0.92	(0.77–2.04)	0.24
CCI	1.97	(0.82–3.13)	0.28
Warm ischemia time *(continuos variable)*	1.15	(0.57–3.24)	0.66
Intermediate-complexity renal masses (Padua 8–9)			
3D virtual model reconstruction *(vs no reconstruction)*	0.89	(0.66–1.41)	0.28
Enucleation *(vs enucleoresection)*	1.11	(0.84–2.01)	0.16
Enucleation *(vs resection)*	0.99	(0.88–1.94)	0.34
CCI	1.81	(0.98–2.33)	0.28
Warm ischemia time *(continuos variable)*	1.23	(0.77–1.49)	0.31
Low-complexity renal masses (Padua 6–7)			
3D virtual model reconstruction *(vs no reconstruction)*	1.01	(0.73–1.14)	0.22
Enucleation *(vs enucleoresection)*	1.04	(0.82–1.22)	0.18
Enucleation *(vs resection)*	1.18	(0.91- 1.31)	0.24
CCI	1.44	(0.87–2.14)	0.11
Warm ischemia time *(continuos variable)*	1.19	(0.91–1.25)	0.19

*CCI* Charlson Comorbidity Index

## Discussion

Today we are facing the era of precision medicine, which is increasingly being applied in the surgical field, including the treatment of oncological pathologies [19]. To achieve this goal, surgery has begun to take advantage of three-dimensionality for the study of the patient’s anatomy, pursuing the creation of a surgery procedure completely tailored to the patient [20, 21]. In the present study we sought to compare the outcomes of two contemporary cohorts of patients submitted to RAPN for renal neoplasm with vs without the use of 3DVMs, after applying propensity-score matching analysis.

Our results highlight some crucial points of discussion. First of all, preoperative patient’s and tumor’s characteristic were comparable between the cohorts in terms of age, comorbidity burden, PADUA score, clinical tumor size and stage. Propensity-score analysis selected two homogeneous populations also in terms of baseline renal function being the greatest majority of patients on CKD stage I and II in both groups. Concerning perioperative outcomes, 3DVM group differed from the control group for the type of clamping adopted during RAPN. In particular, the rate of selective clamping procedures more than doubled when 3DVMs were employed (21% vs 9%; *p* = 0.03). The preference of selective clamping instead of global clamping can be explained considering the role of 3DVMs, providing a more precise and deeper knowledge of the vasculature and allowing the surgeon to optimize ischemic damage with selective clamping performance, as previously demonstrated [22]. Moreover, this finding reinforces what was previously reported by our group in a recent research where we showed how the preoperative surgical strategy of 30 RAPNs has varied when the cases were evaluated separately with 3DVMs and 2D CT-scan images [23]. We found that 26.4% cases had their PADUA score downgraded when switching from 2D CT-scan to 3D virtual model assessment and off-clamp/selective clamping strategy and enucleation resection strategy increased from CT-scan to 3D evaluation. Moreover, with the “rainbow technology”, which is integrated into the 3D rendering, is possible to estimate the different vascular regions of the kidney according to the three-dimensional III order vessels that compose the arterial vascular tree of the organ. This could help to establish the best hilar clamping strategy to balance hypoxic damage and risks of bleeding from the resection bed. We also found a significantly higher rate of enucleative partial nephrectomy (SIB score 0–2) within the 3DVM group (40% vs 29%; *p* = 0.04). This result can be also explained by the anatomical details offered by the 3D reconstruction which allow the surgeon to carefully plan the anatomical excision strategy according to the tumor’s features ultimately maximizing the amount of healthy parenchyma spared. Recent analysis has pointed out the protective impact of enucleation technique on the incidence of PSM [24, 25] and the present study did report similar results. In fact, despite not being significant, we observed a halved rate of PSM in the 3DVM group as compared to the counterpart (3% vs 6%, *p* = 0.05), and this result mirrors the improved ability to follow the tumor pseudocapsule with the assistance of 3DVMs. Whether this difference will translate into better survival outcomes remains to be demonstrated [26]. The overall lower incidence of global ischemia together with the maximization of healthy renal tissue potentially led to improved functional outcomes within patients treated with RAPN with the use of 3DVM. One-year median creatinine serum level (1.2 [IQR 1.1–1.4] vs 1.6 [1.1–1.8]) and eGFR (64.6 [56.2–74.1] vs 52.3 [49.2–74.1]) performed better (*p* < 0.05) in this cohort of patients. Moreover, a significant lower percentage (17% vs 27%, *p* = 0.03) of patients in this group had a clinically meaningful eGFR drop, given by a loss > 30% of their eGFR values. The impact on 3DVMs on functional outcomes following RAPN has recently gained attention in the scientific community with contrasting findings [27]. Porpiglia and his group demonstrated that surgeries assisted by 3DVMs had improved functional preservation as compared to a control group, showing that 3D reconstruction was the only protective factor against a significant functional damage [10]. Recently, another study pointed out the positive impact carried by 3DVMs on 12-month functional preservation as compared to a control series [9]. In particular, after applied PSMA, the authors showed a significantly lower rate of on-clamp procedures in 3DVMs group (7% vs 91%, *p* < 0.001) attributing to this difference the main impact on functional preservation. Our study findings are fully aligned with those reported by Porpiglia and Bernhard [9, 10] but our peculiarity lies on reporting also different tumor excision techniques and how these procedures are affected by the adoption of 3DVMs. Interestingly, a different message emerged from a systematic review and meta-analysis conducted among comparative studies where no difference was observed in terms of eGFR for RAPN carried on with/without 3DVMs [28]. We also harbored skepticism on the unselected adoption of such technology when performing nephrons-sparing surgery, as mentioned in previous publications [29, 30]. As such, we conducted a sensitivity analysis on different PADUA risk-categories (low-risk PADUA 6–7; intermediate-risk PADUA 8–9; high-risk PADUA ≥ 10) showing that the functional benefit was observed only the high-risk category masses (Fig. 1), as confirmed also at the multivariable model (Table 3). The lower impact of 3DVM for low- and intermediate-complexity lesions is explainable considering the lower impact of the surgery on the renal function in these kinds of tumors (more exophytic and smaller). Finally, regarding perioperative risk profile and oncological outcomes both cohorts showed comparable outcomes. RAPN performed with/without 3DVM did not differ in terms of operative and ischemia time, estimated blood loss, complication and local recurrence rate.

Despite its interesting results, some limitations of the present study have to be declared, starting from the absence of randomization and the duration of follow-up. Moreover, kidney function was assessed by creatinine and eGFR levels without including renal scan evaluation. Finally, considering that this data came from a referral center for robotic nephrons-sparing surgery, the results might be poorly translated in all healthcare settings. Nevertheless, this study is the first, to the best of our knowledge, demonstrating the impact of 3DVMs on perioperative and functional outcomes in patients treated with RAPN after applying PSMA and reporting different tumor excision techniques. A cost-effectiveness analysis of such technology is warranted to support its application in daily practice.

## Conclusions

RAPN performed with the use of 3DVM assistance resulted in lower incidence of global ischemia and higher rate of enucleations, as compared to a control group. These outcomes ultimately led to enhanced 12-month functional preservation in this cohort of patients, but this result was confirmed only in high-risk renal masses (PADUA ≥ 10). In light of the potential key role in the daily practice of 3D reconstructions, future prospective randomized studies are needed. It worth underlining that costs of this technology could limit its diffusion in the daily practice.

## Supplementary Information

Below is the link to the electronic supplementary material.

Supplementary file1 (DOCX 15 kb)
