# Supplementary material for: Three-dimensional virtual model for robot-assisted partial nephrectomy: a propensity-score matching analysis with a contemporary control group
Source: World J Urol. 2024 May 20;42(1):338. doi: 10.1007/s00345-024-05043-9 (PMC11106151; doi:10.1007/s00345-024-05043-9)
Supplement: Supplementary file 1 — Supplementary file1 (DOCX 15 kb) [file 345_2024_5043_MOESM1_ESM.docx]

Appendix to the manuscript

1. **Description of the Propensity-score matching analysis screening process and implication on the study findings**

We generated a 1:1 propensity score–matched cohort (nearest-neighbour PSMA using a caliper width of 0.1 of the standard deviation of the logit of the propensity score) to select two homogeneous study groups. Matching variables represented Charlson Comorbidity Index (CCI), PADUA score, and baseline creatinine serum level. Finally, 100 cases for each group were selected. From 3DVM cohort, 52 cases were excluded from the analysis due to inadequate matching variables or because they exhibited exclusion criteria. Excluded cases were solitary kidney patients (9, 17.3%), patients with chronic kidney disease stage >4 (12, 23.0%), patients with enlarged lymph nodes (9, 17.3%), presenting multiple (5, 9.6%) or bilateral (6, 11.5%) tumors. Moreover, 11 (21.3%) patients presented a non-negligible comorbidity burden (CCI >6) and did not match with the counterpart group. The exclusion of such patients reflects the limit of PSM analysis, which from one side selects two comparable population groups, on the other side makes no claim to remove the effects of confounding due to unmeasured covariates. Indeed, a large sample size is required for PSM analysis, as it discards some subjects who are not matched, reducing the statistical power of the analysis. Additionally, imperfect matching can also lead to bias in PSM analysis, as it may not account for all the relevant differences between the matched pairs. For these reasons, findings from observational studies must be interpreted with care given this potential for residual confounding (a limitation shared by regression adjustment).
